# Pediatric surgery in India: Then and now

**DOI:** 10.4103/0971-9261.54810

**Published:** 2009

**Authors:** I. C. Pathak

**Affiliations:** Emeritus Professor of Pediatric Surgery and Former Director, Post Graduate Institute of Medical Education and Research, Chandigarh, India

Surgery of children has been practiced since times immemorial. It is, however, only after the second World War that it was recognized as an independent identity in the USA and United Kingdom. The rapid strides the specialty made in the west during the last six decades is, therefore, astonishing. It has been likened to the evolution of an oak tree from an acorn, growing from humble beginnings to a well recognized surgical specialty.

Pediatric surgery as a specialty is about 45 years old in India. In the fifties of the last century, there were only three general surgeons who confined their work exclusively to pediatric surgery. They were Prof. U.C. Chakraborty at Calcutta Medical College, Dr. Raman Nair at Trivandrum Medical College, and Prof. D. Anjaneyulu at Niloufer Hospital affiliated to Osmania Medical College at Hyderabad.

The fifties were also the years of beginning of my own surgical career. Having graduated from Medical College, Amritsar, I was lucky to be appointed a Surgical Registrar to the then doyen of Surgery Prof. S.S. Anand at Medical College Hospital, Amritsar, in the mid-fifties. Children's surgery at that point of time was chiefly confined to herniotomy, suprapubic lithotomy, appendectomy and drainage of empyema. Most of the general surgeons in those days were not interested or indifferent to the management of severe congenital anomalies. At Amritsar, in the mid-fifties, babies suffering from esophageal atresia, abdominal wall defects and other severe congenital anomalies were usually sent home without surgical treatment for lack of expertise. I believe most of the other Medical Institutes had similar practice at that time. It is not surprising, therefore, that many young surgeons in the late fifties and early sixties went abroad for higher training in pediatric surgery as there were no such training centers in the country. I was fortunate to get a Colombo Plan Scholarship for higher training at Hospital for Sick Children, Great Ormond Street, London, in 1962.

In early and mid-sixties after varying periods of training abroad, young surgeons returned home with great hopes of settling down to practice the new specialty. Many of them were soon disillusioned. There was a stiff resistance from the general surgeons who felt threatened as their specialty had already fragmented by the new specialties of cardiac and neurosurgery, urology and plastic surgery and could not accept further fragmentation.

Two very significant developments took place at this point of time in the development of our specialty. One was the establishment of an independent pediatric surgery section within the frame work of Association of Surgeons of India in December, 1964. The Indian Association of Pediatric Surgeons (IAPS) established in 1965, as a section of Association of Surgeons of India (ASI) separated from its parent body and became an independent Association in 1994. Since then, the IAPS has grown from a humble beginning of 15 in 1965 to the present strength of about 1000 members.

The institution of Postgraduate training leading to a degree in the specialty had a strong impact in the development of the specialty in the country. The Madras University was the first in the country to institute such a course and the first M.Ch. degree was awarded in 1968. Gradually, other Universities/Institutions, including the Post Graduate Medical Institute at Chandigarh, followed suit and by the end of 1972, as many as 7 Institutes were awarding M.Ch degree in pediatric surgery. The result was that the flow of surgeons going abroad for higher training in pediatric surgery dried up. Till 1998, as many as 500 pediatric surgeons had been trained at various centers in the country. At PGI, Chandigarh, we have trained 115 pediatric surgeons so far. At present, I believe there are 23 such higher centers in the country, adding 30-40 pediatric surgeons each year.

It is difficult to assess the progress made by our specialty during the last four decades as there is hardly any published data on the long-term results of management of severe anomalies studied prospectively. In the fifties and early sixties of the last century, mortality was high in neonates with severe congenital malformations. It was only in the mid-sixties when pediatric surgery became an accepted specialty and there was improved success in surgical cases of esophageal atresia, congenital diaphragmatic hernia, atresia of small gut and abdominal wall defects.

A study designed to assess the status of pediatric surgery in India published in 2002 by Dr. D.K. Gupta and his associates from AIIMS on the infrastructure, personnel, survival rates and publication from Institutes imparting postgraduate training is not very encouraging. As the authors stated that this is only a preliminary study, their recommendation to form a national database of neonatal surgery is welcome and should be followed up.

The current status of pediatric surgery in the country appears to be heartening. There has been a visible change in the infrastructure of our intensive care units in the major pediatric surgery centers of the country and also in big corporate hospitals. In the neonatal surgery intensive care unit at Advanced Pediatric Center at P.G.I., Chandigarh, there are 30 beds with 15 ventilators. Neonatal surgery admissions, which 25 years ago used to be about 10% of our emergency surgical admissions, has gone up to nearly 50 percent now. Esophageal atresia, which was sporadic in those days, is the most common pediatric surgical emergency at P.G.I. these days. Anorectal malformations were the commonest neonatal emergency admission then.

Besides improvement in the intensive care units, there has been a dramatic change in the practice of pediatric surgery during the last decade. This has been the result of newer diagnostic tools, newer imaging techniques, use of interventional radiology, development of ambulatory day care surgery, and change of surgical management.

The change of management of surgical diseases of children has been one of the most significant developments in the recent past and bodes well for the progress and development of pediatric surgery in the country.

The greatest innovation which has found wide acceptance is the use of minimal invasive surgery with the help of laparoscope for an increasing number of surgical conditions. Laparoscopic surgery has been widely practiced in adults in our country for the last 2 decades. It took some time for the pediatric surgeons to undertake laparoscopic procedures and as a result of improvement in instrumentations, digital videoscopy and an increasing number of training centers, many pediatric surgeons are opting for this technique in an increasingly wide field of diseases.

I would now like to present some of the significant contributions from the Department of Pediatric Surgery at PGI during the last 4 decades.

## ANORECTAL MALFORMATIONS

Prone cross-table lateral view – An alternative to the invertogram in imperforate anus (published in February 1983) by K.L.N. Rao and associates provides equal or some times better information compared to the invertogram for demonstration of the level of atresia. An easy positioning, better co-operation of the patients, elimination of the affect of gravity and better visualization of the rectal gas shadow are the advantages of the new technique.

In 1972, we reported a peculiar variant of anorectal malformation titled “short colon associated with imperforate anus” from P.G.I., which had not been reported from India earlier. This is a condition in which the entire colon was replaced by a dilated sac which had a wide fistulous communication with the urinary bladder in boys and with the vagina in girls. There was a characteristic radiologic appearance. Nearly the entire abdomen was filled with a single air and fluid level.

By 1984 – we had an experience of a large number of similar cases and noticed that in few cases, some part of the colon beyond the caecum was normal and proposed a classification of the types, and the large sac-like transformation of the colon was in fact more of a pouch, we suggested that the anomaly be designated as a “pouch colon syndrome.” By the year 2000, we had seen a total of 137 patients. Vast majority of cases are reported from North India, like Chandigarh, Patiala, Delhi, Lucknow and Varanasi.

A prospective study was carried out at the Department of Pediatric Surgery, Chandigarh, by Menon and Rao in cases of anovestibular fistula in female babies since 1997 and published in 2007. Posterior sagittal anorectoplasty without a colostomy cover has shown commendable results. Anal dilatation is not required after surgery.

## VESICOURETERAL REFLUX

Management of primary vesicoureteral reflux (VUR) is a controversial subject. A prospective study on the long-term effects of surgical and conservative treatment on renal status and somatic growth with severe VUR at the Department of Pediatric Surgery (K.L.N. Rao and associates) from 1996 to 2003 showed that 15% of grade IV–V reflux and all the cases of grade I–III reflux resolved completely with conservative treatment with significant weight gain. Surgery and resolution of reflux also improved the growth parameters. A new treatment of VUR by an endoscopic injection of Deflux is a simple and common procedure now, thereby avoiding surgery in most cases.

## PELVI URETERIC JUNCTION OBSTRUCTION

Hydronephrosis caused by pelviureteric junction (UPJ) obstruction is one of the commonest clinical problems in pediatric urology. A study carried out by the Department of Pediatric Surgery, P.G.I., and published in 2003 on 30 cases (K.L.N. Rao) showed that the histologic extent of the abnormally innervated ureteral segment is longer than the visible, constricted segment of the ureter. It is recommended that the ureter should be excised at least 8 mm or more beyond the visible lower length of constricted segment to completely eliminate the neurologically obstructed ureteric segment. This study represents the first attempt at providing guidance regarding the extent of ureteral resection based on basic research founded on neurogenic theory of U.P.J. obstruction.

## EXTRA HEPATIC PORTAL HYPERTENSION

Extrahepatic portal hypertension (EHPH) is a common cause of upper gastrointestinal bleeding in children and infants. Portal hypertension and bleeding esophageal varices can be managed both medically and surgically. Several centers have reported excellent results with sclerotherapy alone.

Surgical management as a one-time treatment of EHPH is a more feasible alternative compared with repeated sclerotherapy and a life long follow up. Sclerotherapy is also not capable of treating hypersplenism, growth retardation, and portal hypertension colopathy. Shunt surgery for EHPH not only offers the patient a one-time elective procedure but also takes care of the above problems.

A comparative prospective study of the three modalities of surgical treatment for EHPH showed improvement in growth parameters and quality of life after elective surgery on 30 children from the Department of Pediatric Surgery, Chandigarh. It has been shown that all procedures were comparable in the fall of portal pressure after surgery. Central splenorenal shunt (CSS) after splenectomy was found to be useful in cases with large spleen, severe hypersplenism and a suitable splenic vein. Side-to-side lienorenal shunt without splenectomy (SSLR) is suitable when there is mild splenomegaly and a shuntable vein. Splenectomy and devascularization (SGD) is the choice when there is no shuntable vein.

## ESOPHAGEAL ATRESIA

Pure esophageal atresia and esophageal atresia with a long gap form the most difficult subset of babies born with esophageal atresia or tracheo-esophageal fistula. The best modality of esophageal replacement remains controversial. For esophageal replacement, in addition to colon, gastric tubes, and gastric pull up, a new technique of a tube created from the fundus of stomach to be used as a replacement has been developed at P.G.I., Chandigarh on 2003 (K.L.N. Rao and associates) I believe that nearly 30 babies have benefited from this procedure at P.G.I.

## FUTURE

In the not too distant future, I see three very exciting prospects for pediatric surgery in the country. One, there will be a far greater use of minimal access laparoscopic surgery in the pediatric age group than at present, and two, robot-controlled surgery, already practiced in the country in adults in a few centers currently, would be introduced in the pediatric population in the near future enhancing surgical precision, Third, organ transplantation, already well established in adult surgery, would attract participation by pediatric surgeons in liver and renal transplants in the near future.

## CONCLUSION

Great advances may follow in the future; the basic principles of surgery would continue to guide us in the care of children. I may, therefore, be permitted to conclude this address by quoting Willis Potts – one of the pioneers of pediatric surgery in North America, highlighting these very principles from the preface of his book “Surgeon and the Child” published in 1959 – dedicating it to the infant who has the great misfortune of being born with a serious deformity saying that “if this infant could speak, it would beg imploringly of the Surgeon, please exercise the greatest gentleness with my miniature tissues and try to correct the deformity at the first operation. Give me blood and the proper amount of fluid and electrolytes; and plenty of oxygen to the anesthesia and I will show that I can tolerate terrific amount of surgery. You will be surprised at the speed of my recovery and I shall be always grateful to you.”

